# Neurexin 3 transmembrane and soluble isoform expression and splicing haplotype are associated with neuron inflammasome and Alzheimer’s disease

**DOI:** 10.1186/s13195-019-0475-2

**Published:** 2019-03-21

**Authors:** Akitoyo Hishimoto, Olga Pletnikova, Doyle Lu Lang, Juan C. Troncoso, Josephine M. Egan, Qing-Rong Liu

**Affiliations:** 1 Department of Psychiatry, Kobe University Graduate School of Medicine, 7-5-1 Kusunoki-Cho, Chuo-Ku, Kobe, 650-0017 Japan; 2 Departments of Pathology, Neuropathology Division, Johns Hopkins University School of Medicine, 600 North Wolfe Street, Baltimore, MD 21205 USA; 3 Lab of Clinical Investigation, NIA-NIH, 251 Bayview Blvd, Baltimore, MD 21224 USA

**Keywords:** Alzheimer’s disease, Neurexins, Endocannabinoids, Apolipoprotein E, Alternative splicing

## Abstract

**Background:**

Synaptic damage precedes neuron death in Alzheimer’s disease (AD). Neurexins, *NRXN1*, *NRXN2,* and *NRXN3*, are presynaptic adhesion molecules that specify neuron synapses and regulate neurotransmitter release. Neurexins and postsynaptic neuroligins interact with amyloid beta oligomer (AβO) deposits in damaged synapses. *NRXN3* gene variants have been associated with autism, addiction, and schizophrenia, however, not fully investigated in Alzheimer’s disease. In the present study, we investigated an AD association of a 3′-splicing allele of rs8019381 that produces altered expression of transmembrane or soluble *NRXN3* isoforms.

**Methods:**

We carried out RT-PCR (reverse transcription polymerase chain reaction), PCR-RFLP (PCR and restriction fragment length polymorphism), Sanger sequencing, and in situ hybridization (ISH) assays for *NRXN3* neuron expression and genotyping. Genetic associations were analyzed by *χ*^2^ tests, and ISH signals were analyzed by FISH v1.0 module of Indica Labs HALO software.

**Results:**

We previously identified a functional haplotype in the 3′ region of neurexin 3 (*NRXN3*) gene that alters the expression ratios between *NRXN3* transmembrane and soluble isoforms. In this study, we found that expression and ratio of transmembrane and soluble *NRXN3* isoforms were reduced in AD postmortem brains and inversely correlated with inflammasome component *NLRP3* in AD brain regions. The splicing haplotype related to the transmembrane and soluble *NRXN3* expression was associated with AD samples with *P* = 6.3 × 10^−5^ (*odds ratio = 2.48*) and interacted with *APOE* genotypes.

**Conclusions:**

We found that the SNP rs8019381 of *NRXN3* that is located adjacent to splicing site #5 (SS#5) interacts with the *APOE* ε4 haplotype and alters *NRXN3* transmembrane or soluble isoform expression in AD postmortem cortex. Dysregulation of presynaptic *NRXN3* expression and splicing might increase neuron inflammation in AD brain.

## Background

Non-familial and late-onset Alzheimer’s disease (AD) is a common cause of dementia in the elderly. Emphases on classical AD neuropathological features, Aβ neuritic plaques (Aβ-NPs), neurofibrillary tangles (NFTs), and neuropil threads are increasingly acknowledged to be accompanied by disrupted synaptic contacts and impaired glutamatergic neurotransmission [1, 2]. While the ε4 allele of apolipoprotein E (*APOE*) gene makes a large contribution to the genetic bases of interindividual differences in vulnerability to AD, the sizable genetic influences that remain after accounting for *APOE* are likely to arise from polygenic and/or rarer variants that each makes modest contributions to overall disease vulnerability.

Diffuse Aβ fibrillar plaques are often observed in postmortem human brains with normal cognitive function [3–6]. Pathological Aβ plaque formation around synapses with AβO deposit correlates with memory loss and synapse dysfunction [7, 8]. Neurexins were discovered as α-latrotoxin (venom of black widow spider) receptors [9] and function as presynaptic cell adhesion molecules [10] that help to regulate the release of neurotransmitters, specify, and stabilize classical synapses, including the glutamatergic synapses that provide a focus for research in AD [1, 2]. Neurexin genes are among the largest genes (greater than one million base pairs) in the human genome, and the three mammalian neurexin genes *NRXN1*, *NRXN2,* and *NRXN3* each display differential splicing events that provide thousands of neurexin isoforms on a background of longer α-neurexin and shorter β-neurexin that arise from the use of alternative promoters [11]. The larger α-neurexins contain three EGF-like (epidermal growth factor) domains each of which flanked by two LNS (lamin-neurexin-sex hormone-binding globulin) domains, a single transmembrane domain, and intracellular PDZ (*PSD95*-*Dlg1*-*Zo1*) domain that interact with intrasynaptic proteins [10, 12]. Specifically, α-neurexins are coupled to presynaptic calcium channels to regulate neurotransmitter release [13] and interact with postsynaptic neuroligins, leucine-rich repeat transmembrane proteins (*LRRTM*s), calsyntenins (*CLSTN*), α-dystroglycan (*DAG1*), GABA_A_-receptors (*GABRA*s), latrophilins (*ADGRL*s), cerebellin (*CBLN*)-glutamate dehydrogenase (*GLUD*) complexes, synaptic cleft secreted neurexophilins (*NXPH*s), and intracellular PDZ-binding proteins [14, 15]. Neurexins’ intracellular PDZ domains can bind to *MINT1*, *MINT2*, and *CASK* proteins [16, 17] that themselves bind to and stabilize the transmembrane form of amyloid precursor protein (*APP*) [18]. *MINT1* and *MINT2* are adaptor proteins that complex with conserved motifs in *APP*’s C-terminal region to stabilize *APP* transmembrane forms and reduce secretion of pathogenic Aβ cleavage products [19]. It is thus even possible that *NRXN*s-*MINT*s interaction complexes could alter *APP* protein processing. Soluble or secreted α-*NRXN3* is produced by including extra exon 23 with four different intra-exonal spliced sites that encode four premature stop codons that abolishes the transmembrane and intracellular PDZ domains [20, 21]. The smaller β-neurexin contains one LNS domain (no EGF domain), a transmembrane domain, and an intracellular PDZ domain. The β-neurexin acts as a brake for endocannabinoid 2-AG (2-arachidonoylglycerol) synthesis that retrogradely regulates presynaptic cannabinoid receptor 1 (CB1R)-mediated depolarization-induced suppression of excitation on AMPA and NMDA receptors that are involved in excitatory postsynaptic currents (EPSCs) [22]. *APP* cleavage enzymes of α- and γ-secretases can process β-*NRXN3* into an N-terminal extracellular domain (80 kDa) and a C-terminal intracellular domain (12 kDa). The enzymatic activities are altered by several single nucleotide polymorphisms (SNPs) of γ-secretase subunit presenilin 1 (*PSEN1*) that contribute to early-onset forms of familial AD [23]. Recent data identify roles of neurexin isoforms in several complex neuropsychiatric phenotypes that include autism [24–26], addiction [20, 27, 28], and schizophrenia [29, 30].

*NRXN3* mRNA is the second most reduced gene after vacuolar H^+^-ATPase subunit gene *ATP6V1E1* in AD hippocampus identified by bioinformatic analysis of AD and aging Gene Expression Omnibus (GEO) databases [31, 32]. We have identified 3′ region of *NRXN3* haplotypes that are tagged by alleles of the SNP rs8019381, which is located near the end of *NRXN3*’s exon 23 at a key splicing site [20]. Alleles of rs8019381 tagged *NRXN3* produce the splice variants that include or exclude exon 23 coding for a single transmembrane domain; thus, transmembrane or soluble *NRXN3* isoforms are transcribed and translated, respectively [20]. *NRXN3* is expressed in neurons in brain regions that are implicated in mnemonic processes and in dementia-associated AD pathologies. For example, *NRXN3* is expressed in the cerebral cortex and in the hippocampus that contains AD-related senile plaques and neurofibrillary tangles [33, 34]. Differences in the properties of synapses in these regions could alter brain connectivity, and the altered ratio of transmembrane and soluble *NRXN3* isoforms could lead to pathological AβΟ accumulation at synapses.

We have thus characterized the patterns of expression of total *NRXN3*, α-*NRXN3* and β-*NRXN3*, and four *NRXN3* transmembrane and soluble splice variants in mRNAs extracted from postmortem middle frontal gyrus from pathologically confirmed AD and control individuals. We have sought AD-related differences in frequencies of *NRXN3* haplotypes and tested whether the *NRXN3* associations are dependent on the *APOE* genotype. Finally, we have found evidence for *NRXN3* association and interaction with *APOE* genotypes in previously reported genome-wide association datasets and for *NRXN3* inverse correlation with inflammasome component *NLRP3* in neurons of the AD hippocampus and cortex. Taken together, these data support contributions for common human *NRXN3* haplotypes and altered *NRNX3* transmembrane and soluble isoform expression in AD brain.

## Methods

### Subjects: human samples

Middle frontal gyrus postmortem brain samples of 121 European-American AD (38 men and 83 women with mean age 80.3 ± 9.6) were obtained from the Division of Neuropathology, the Department of Pathology, the Johns Hopkins University School of Medicine (JHUSOMI), whose diagnoses were all confirmed by autopsy [35].

Additional European-American comparison groups comprising 349 subjects were examined to document the distribution of *NRXN3* polymorphism in the general American Caucasian population of the USA. One hundred sixty samples (107 men and 53 women with mean age 36.1 ± 16.2) were obtained from Maryland Brain Bank (UMD) whose geographical location is close to that of AD subject collection. One hundred eighty-nine unrelated subjects (42 men and 147 women with mean age 51.2 ± 14.9) were selected from pedigrees from the Collaborative Study on the Genetics of Alcoholism (COGA) [36]. We explored mRNA levels of *NRXN3* isoforms in middle frontal gyrus postmortem samples from 58 pathologically confirmed AD subjects and 48 control subjects. We also explored the association between this *NRXN3* haplotype and AD with 121 pathologically confirmed AD subjects and 349 control subjects.

### RNA isolation and cDNA synthesis

Total RNA was extracted from the larger sized middle frontal gyrus samples available from 58 of the autopsy-confirmed European-American AD patients and 48 normal individuals dying without neurological disease (40 females/18 males, mean age ± SD; 81.2 ± 10.0, PMI; 11.4 ± 6.4 from JHMI and 11 females/37 males, mean age ± SD; 47.6 ± 19.7, PMI; 11.9 ± 6.0 from JHMI and UMD) using Trizol (Thermo Fisher, Waltham, MA) protocol. Single-strand cDNA was synthesized from total RNA using SuperScript™ III One-Step RT-PCR System (Invitrogen, Carlsbad, CA, USA). Electrophoresis of all RNAs used for quantitative PCR revealed sharp 18S and 28S ribosomal RNA bands; four samples with evidence for RNA degradation were eliminated on this basis.

### Quantitative RT-PCR

For quantitative real-time PCR assessments of *NRXN3* mRNAs, isoform-specific primers and minor groove-binding (MGB) TaqMan probes were designed using Primer Express Software (Table 1), common *NRXN3* TaqMan probe (Hs01028186_m1), and endogenous control glyceraldehyde-3-phosphate dehydrogenase (*GAPDH*, Vic-labeled) which were ordered from Thermo Fisher (Cat#4326317E, Thermo Fisher Scientific, Waltham, MA, USA). The relative fold change is calculated using the formula 2^(−△△*C*_*t*_).

**Table 1:** Real-time PCR primers and MGB Fam-TaqMan probes for *NRXN3* isoforms

*NRXN3*	Forward primers	Reverse primers	MGB probes
Hs01028186_m1	NRXN3 TaqMan gene expression assay		
ex 22a24a	TGATCTTGTTTCATCTGCTGAATG	AAGGTGCACGAGTAGCAATAG	CCGAGTACAGGAGGTG
ex 22a24b	TGATCTTGTTTCATCTGCTGAATG	TGCTTTGTAGCCACCTTCGA	CCGAGTACAGATAAGAGTC
ex 22a24c	TGATCTTGTTTCATCTGCTGAATG	CCCGGAACCCGTCTGATT	CCGAGTACAGCAAAC
ex 22a23a	AGATGATCTTGTTTCATCTGCTGAA	CGGAGTGATCTAGCTGCATTAGAG	CCGAGTACAGCCAGAAG
ex α1-2	GACATACAGACAGATCCCAAATCTTC	TCATGGTGCGGCCAGAA	AACTGGAAAGGTCTTTTC
ex β1-18	TTCCCCTGTTTCCCTTCGA	GCCCACCACTTTTCCCAAA	AGGACACGCTGGCG

### Genetic testing

Genomic DNA was extracted from blood or tissue samples from the individuals noted in “human samples” above. DNA from most AD and control samples was extracted from the middle frontal gyrus brain tissues using Qiagen genomic DNA kits. Genomic DNA for other subjects was extracted from peripheral leukocytes as previously described [37]. *APOE* polymorphisms were genotyped using PCR-RFLP assays as described [38]. The rs8019381 SNP was genotyped by direct Sanger sequencing as described [20].

### RNAscope in situ hybridization (ISH)

Human postmortem hippocampus (1 control sample of Braak 0 and 3 AD samples of Braak 6) and middle temporal gyrus (2 control samples of each Braak 0, 1, and 2 and 2 AD samples of each Braak 4, 5, and 6) were used for triplex fluorescent ISH. Human RNAscope ISH probes were ordered from Advanced Cell Diagnostics Inc. (ACD, Hayward, CA, USA) for *NRXN3* in C2 channel (20 ZZ pairs targeted region 1095–2035 of NM_001105250.2; Cat No. 525431-C2), *NLRP3* in C1 channel (30 ZZ pairs targeted region 2627–4008 of NM_004895.4; Cat No. 478021), and *NEUN*/*RBFOX3* in C3 channel (20 ZZ pairs target region 720–2217 of NM_001082575.2; Cat No. 415591-C3). The positive control probes (Cat No. 320868) were *POLR2A* (C1 channel), *PPIB* (C2 channel), and *UBC* (C3 channel). The negative control probe was bacterium (*Bacillus subtilis*) gene *DapB* (Cat No. 320871). The cryostat sectioning of postmortem human brain samples, fixation, protease pretreatment, probe hybridization, pre-amplification, amplification, horseradish peroxidase reaction, and fluorescent labeling steps were described previously [39]. Zeiss LSM 880 confocal microscope was used to image fluorescent labeling. Amplification × 20 images (two to three images for each brain sample) were analyzed by FISH v1.0 module included in HALO software with RNAscope ISH setting (Indica Labs, Corrales, NM, USA). The H-score [Σ_bin0–4_ (ACD score or bin number × percentage of cells per bin)] were used to calculate mRNA expression for each probe based on the minimum intensity threshold (a value between 0 and 400). 

### Statistical analysis

Genetic associations were analyzed by χ^2^ tests. Deviations from Hardy-Weinberg equilibrium (HWE) were examined by *χ*^2^ test with *P* < 0.05 as a deviation from HWE. Correction for multiple testing was not applied because of the a priori reason to focus on rs8019381 in this study. Power analyses used the program PS v2.1.31 [40]. Comparison of the ages in AD between the rs8019381 SNP genotype groups was analyzed using ANOVA. Logistic regression analysis was also applied using phenotype as the dependent variable, and the age, gender, APOE ε4 allele, and rs8019381 genotypes as the independent variables. Statistical analyses of mRNA expression RT-PCR and ISH data were performed using PRISM (GraphPad Software, CA, USA) software. Differences in the mRNA expression levels based on phenotype (control vs AD or genotype CC vs CT and TT) were examined using two-tailed Mann-Whitney tests. Two-way ANOVA and two-tailed/unpaired Student’s *t* test using H-scores of ISH intensities were tested for any significant differences between *NRXN3* and *NLRP3* expressions in different Braak stages of MTG and HIP samples. Linear regression of H-scores of each ISH probe was used to fit straight lines through control and AD data sets with different Braak staining stages and statistically calculated for any significant differences. *P* < 0.05 was considered significant for comparisons of expression levels. Spearman’s rank correlation coefficient analyses were used to assess the contributions of age, sex, and postmortem interval to the mRNA expression levels of each splice variant.

## Results

### α-*NRXN3* and β-*NRXN3* mRNA expression in AD middle frontal gyrus

**Fig. 1:**
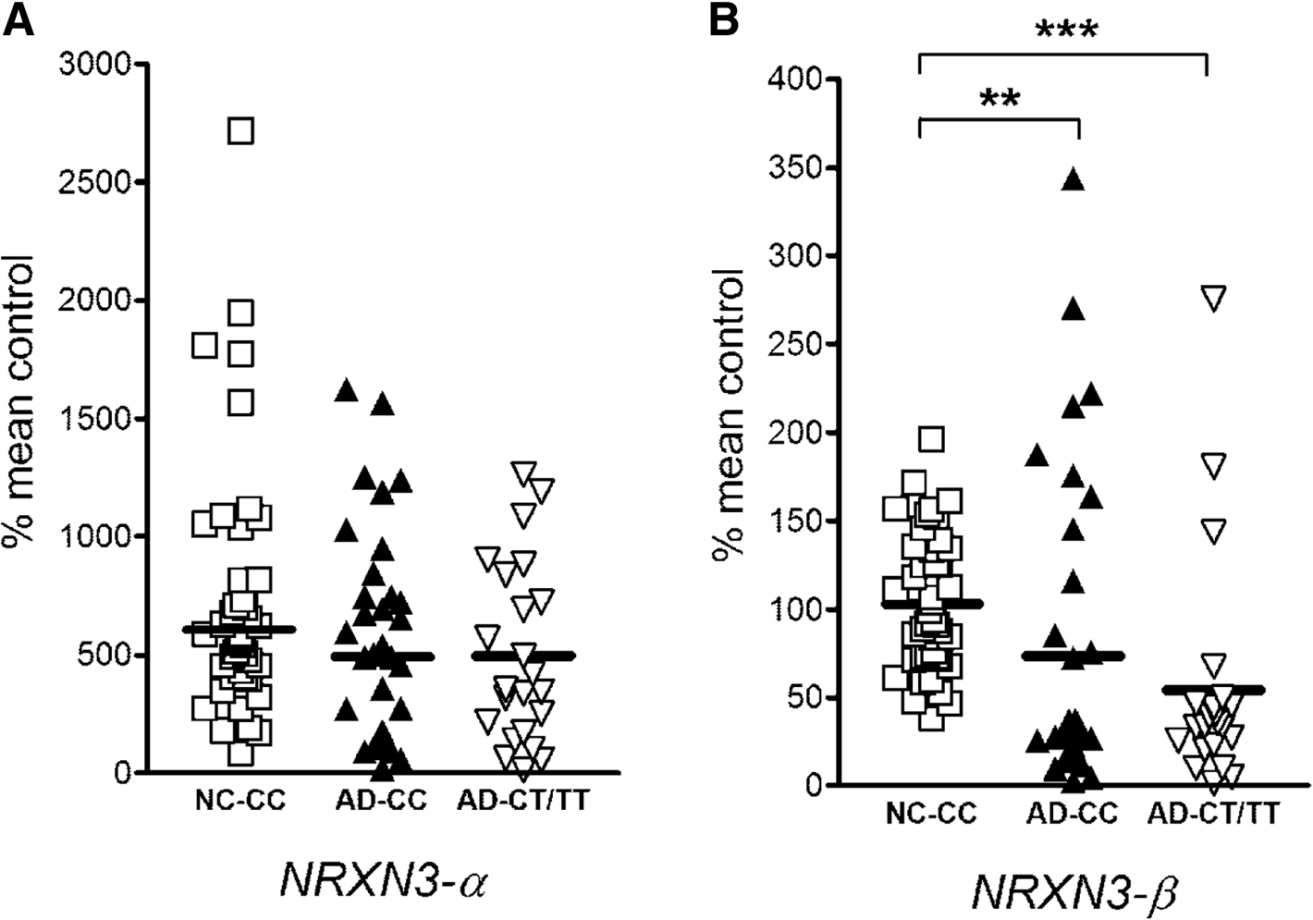
α-*NRXN3* (**a**) and β-*NRXN3* (**b**) mRNA expressions in the middle frontal gyrus of control individuals (*n* = 44 individuals with rs8019381 CC genotype) and AD patients (*n* = 35 individuals with rs8019381 CC genotype and *n* = 23 individuals with CT or TT genotypes). Relative levels of each mRNA expression were obtained after normalization to *GAPDH*. Then, percentile change is obtained respective to an average of *NRXN3-β* mRNA in control individuals with CC genotype. **P < 0.001, ***P < 0.0001, calculated using two-tailed Mann-Whitney tests

**Fig. 2:**
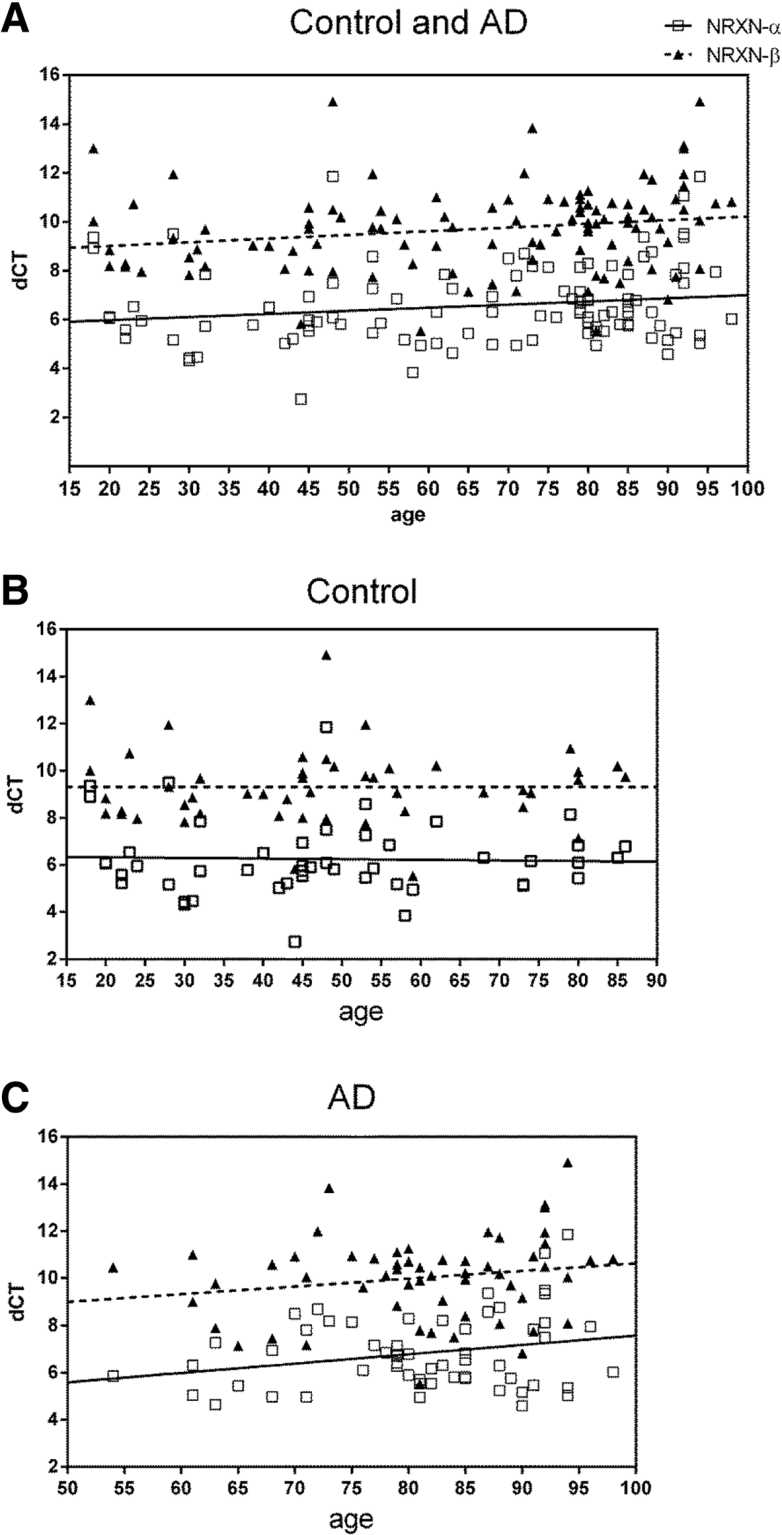
**a** Correlation between age and either α-*NRXN3* or β-*NRXN3* mRNA expression levels in the control and AD combined groups (*P =* 0.0473, Spearman *r* = 0.193 and *P =* 0.0061, Spearman *r* = 0.2648, respectively). **b** Correlation between α-*NRXN3* and β-*NRXN3* mRNA expression levels in the control group and age (*P* = 0.787, Spearman *r* = 0.040 and *p* = 0.386, Spearman *r* = 0.128, respectively), and **c** in the AD group and age (*P* = 0.253, Spearman *r* = 0.153 and *P* = 0.186, Spearman *r* = 0.176, respectively). Δ*C*_*t*_ values of each isoform expression were obtained after normalization to *C*_*t*_ values of *GAPDH*

We compared expression of α-*NRXN3* and β-*NRXN3* in mRNAs extracted from the middle frontal cortices of human postmortem brain samples of AD and controls with different “splicing site 5 (SS#5)” *NRXN3* haplotypes defined by alleles of the rs8019381 SNP. Since controls displayed few rs8019381 T alleles, we compared control samples with CC genotypes to AD samples with CC genotype and to AD samples with either one or two T alleles (CT/TT). There were no significant differences between expression of α-*NRXN3* mRNA in CC controls vs AD patients with either CC or CT/TT genotypes (Fig. 1a; two-tailed Mann-Whitney *P* = 0.067 and *P* = 0.127, respectively). By contrast, when compared with control individuals with CC genotypes, β-*NRXN3* mRNA expression levels decreased by 30% and 48% in AD patients with CC and CT/TT genotypes, respectively (Fig. 1b; *P* = 0.0004 and *P* < 0.0001, respectively). We identified a mild significant correlation between age and either α-*NRXN3* or β-*NRXN3* mRNA expression levels in the combined groups (Fig. 2a; *P =* 0.0473, Spearman *r* = 0.193 and *P =* 0.0061, Spearman *r* = 0.2648, respectively). However, we identified neither a trend nor a significant correlation between age and either α-*NRXN3* or β-*NRXN3* mRNA expression levels in the control groups (Fig. 2b; *P =* 0.787, Spearman *r* = 0.040 and *P =* 0.386, Spearman *r* = 0.128, respectively). We also did not identify significant correlations between α-*NRXN3* and β-*NRXN3* mRNA expression levels in the AD group and age (Fig. 2c; *P* = 0.253, Spearman *r* = 0.153 and *P* = 0.186, Spearman *r* = 0.176, respectively). In these AD samples, the positive slope of this regression line does indicate trends toward less *NRXN3* expression in older individuals with AD phenotype. Neither gender nor postmortem intervals were correlated with these expression levels in control or AD groups (data not shown). In the AD subjects, there were no differences in expression of either α-*NRXN3* (*P* = 0.751) nor β-*NRXN3* isoforms (*P* = 0.863) in individuals with haplotypes marked by CC vs CT/TT genotypes.

### Genetic analysis

Table 2 shows the genotype distribution and allele frequency of rs8019381 SNP for AD and control groups. The genotype distributions differed remarkably between the AD and control groups (*χ*^2^ = 15.587, df = 2, *P* = 0.000413). The minor allele frequency of the rs8019381 T allele was significantly greater for the AD group than for the control group (0.157:0.070, respectively; *χ*^2^ = 15.997, df = 1, *P* = 0.0000634). These results correspond to an odds ratio of 2.48 (95% confidence intervals 1.57–3.91) for AD in individuals with this T allele. Based on the observed allele frequency of the rs8019381 SNP, the current samples yielded the power of 0.894 for detecting nominally significant results. There was no significant effect of age on the distribution of rs8019381 genotypes in the AD group (*P* = 0.562 by one-way ANOVA). The rs8019381 genotype distributions also displayed no significant deviation from Hardy-Weinberg equilibrium in either the AD or control groups (data not shown).

**Table 2:** Distribution of the *NRXN3* rs8019381 C/T SNP and *APOE* allele frequencies among the rs8019381 genotypes

Group		Genotype^a^			Allele frequency		*P* value
		CC	CT	TT	C	T	
rs8019381 genotype and allele frequencies^b^							
Control (*n* = 336)		291 (0.866)	43 (0.128)	2 (0.006)	0.930	0.070	Genotype: *P* = 0.00041 (*X*^2^ = 15.6, df = 2) Allele: *P* = 0.000063 (*X*^2^ = 16.0, df = 1)
AD (*n* = 121)		86 (0.711)	32 (0.264)	3 (0.025)	0.843	0.157	
rs8019381 genotypes among *APOE* ε4 non-carriers and *APOE* ε4 carriers							
Control	*APOE* ε4 non-carriers	216 (0.857)	35 (0.139)	1 (0.004)	0.927	0.073	*APOE* ε4 non-carriers VS.ε4 carriers in the AD Genotype: *P* = 0.018 (*X*^2^ = 8.0, df = 2) Allele: *P* = 0.000063 (*X*^2^ = 0.43, df = 1)
	*APOE* ε4 carriers	75 (0.893)	8 (0.095)	1 (0.012)	0.940	0.060	
AD	*APOE* ε4 non-carriers	26 (0.722)	7 (0.195)	3 (0.083)	0.819	0.181	
	*APOE* ε4 carriers	60 (0.706)	25 (0.294)	0 (0.000)	0.853	0.147	

^a^Number of subjects (frequency)

^b^rs8019381: Significant differences were found between the AD and the controls in either the genotype distribution (*χ*^2^ = 15.587, df = 2, *P* = 0.000413) or the allele frequencies (*χ*^2^ = 15.997, df = 1, *P* = 0.0000634)

We next investigated the *APOE* genotypes in these samples and sought possible interactions with the effects of the *NRXN3* haplotypes marked by the rs8019381 SNP (Table 2). As expected, the *APOE* genotype and allele frequency distributions of the AD samples differed significantly from those of the control group (*χ*^2^ = 87.146, df = 5, *P* = 2.671 × 10^−17^ and *χ*^2^ = 92.374, df = 2, *P* = 8.735 × 10^−21^, respectively; Table 2).

When we sought interactions between the rs8019381 SNP and *APOE* genotypes in the AD group, the rs8019381 genotype distributions displayed significant differences between *APOE* ε4 non-carriers and *APOE* ε4 carriers (*χ*^2^ = 8.043, df = 2, *P* = 0.0179). Among AD individuals, rs8019381 TT homozygotes were found only in those who did not carry *APOE* ε4 alleles. This difference provided significance for recessive analysis (comparing CC + CT vs TT) of the differences between AD *APOE* ε4 non-carriers and AD *APOE* ε4 carriers (*χ*^2^ = 6.317, df = 1, *P* = 0.012), though not for analyses of allele frequencies (*χ*^2^ = 0.429, df = 1, *P* = 0.513). These results contrasted with those in control samples, where we found that neither genotype distributions nor allele frequencies were significantly different between *APOE* ε4 non-carriers and *APOE* ε4 carriers (*P* = 0.428 and 0.541 for genotype and allele comparisons, respectively).

Overall, significant associations between the rs8019381 genotypes and AD thus remained in both *APOE* ε4 non-carriers (*P* = 0.000403) and in *APOE* ε4 carriers (*P* = 0.00331). The allele frequencies for the rs8019381 SNP also differed significantly in AD vs control comparisons in both the *APOE* ε4 non-carriers (*P* = 0.00252) and *APOE* ε4 carriers (*P* = 0.00827). We also confirmed a significant effect of rs8019381 polymorphism on the AD phenotype considering for age, gender, and APOE ε4 allele (*P* = 0.00157, Table 3).

**Table 3:** Logistic regression analysis of rs8019381 C/T SNP on the AD phenotype considering for age, gender, and *APOE* ε4 allele

Coefficients of bias-reduced logistic regression				
Variable	Parameter	Standard error	Wald *χ*^2^	*P* value
Intercept	− 13.917	1.528	− 9.107	< 0.0001
Age	0.179	0.020	8.750	< 0.0001
Gender	− 0.051	0.416	− 0.123	0.902
*APOE* ε4 allele^a^	1.691	0.403268658	4.194	0.000033
rs8019381 genotype^b^	3.078	0.968	3.181	0.00157

^a^For *APOE* ε4 allele analyses, ε4 non-carriers were coded as 0 and *APOE* ε4 carriers were coded as 1

^b^For rs8019381 genotype analyses, each SNP was coded as 0 for major allele homozygotes, 0.5 for heterozygotes, and 1 for minor allele homozygotes

As anticipated for loci on distinct chromosomes, *APOE* (19q13) and *NRXN3* (14q24) markers displayed evidence for independent segregation in these samples. Neither the genotype distribution nor allele frequency of rs8019381 SNP was associated with the *APOE* allele frequency among the AD or control groups (*P*_AD_ = 0.061, *P*_CTL_ = 0.850 and *P*_AD_ = 0.600, *P*_CTL_ = 0.283 for genotypic and allelic comparisons, respectively).

### Genetic variation and *NRXN3* SS#5 splice variants in AD

**Fig. 3:**
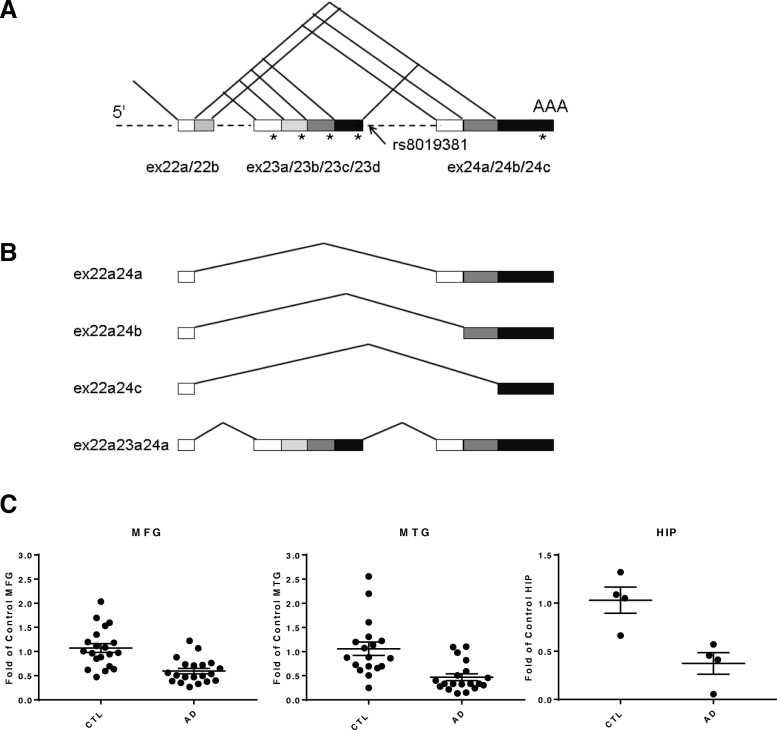
**a** The approximate location of rs8019381 and splicing patterns at the SS#5 of the *NRXN3* gene. The number of potential variants for the splicing site is 30. Exon 24 is the last exon of the gene that codes for the transmembrane region, cytoplasmic domain, and contains 3′UTR. Because any exon 23 isoforms include in-frame stop codons (depicted as asterisks), if it is inserted, soluble α-*NRXN3* and β-*NRXN3* can be produced from this gene. **b** The three major transmembrane isoforms and one major soluble isoform are investigated in the prefrontal cortex in individuals with AD (*n* = 58) and controls (*n* = 48). Each probe for the RT-PCR was designed across both exons. **c** Expression levels of total *NRXN3* mRNA in the middle frontal gyrus (MTG), middle temporal gyrus (MTG), and hippocampus (HIP)

Since the *NRXN3* haplotype tagged by rs8019381 (Fig. 3a) has been associated with altered patterns of expression of *NRXN3* splice variants that encode transmembrane vs soluble isoforms, we evaluated the distributions of these isoforms in control brains, which were virtually all from individuals with CC haplotypes, and in frontal cortex samples of AD brains from CC, CT, and TT individuals. We have previously noted that the predominant *NRXN3* transmembrane isoforms that arise from alternative splicing at SS#5 are exon 22a-24b, exon 22a-24c, and exon 22a-24a, while the predominant soluble isoform comes from exon 22a-23a-24a. We thus assessed the levels of these four isoforms (Fig. 3b).

**Fig. 4:**
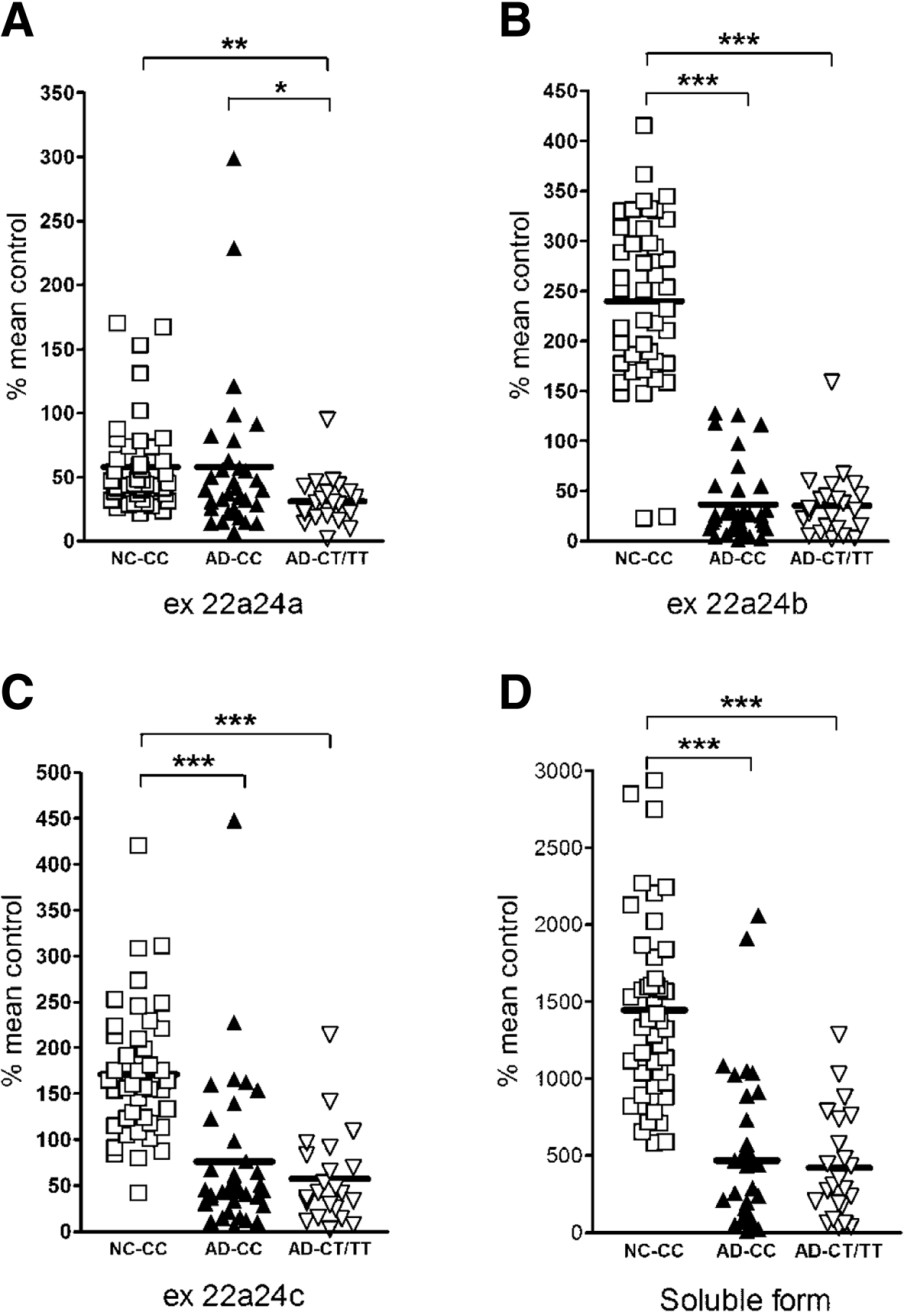
**a**–**c** Expression levels of mRNAs encoding *NRXN3* transmembrane isoforms (exon 22a-24a, exon 22a-24b, and exon 22a-24c variants, respectively) and **d** *NRXN3* soluble isoform (exon 22a-23a variant) in the middle frontal gyrus of control individuals (*n* = 44 individuals with rs8019381 CC genotype) and AD patients (*n* = 35 individuals with rs8019381 CC genotype and *n* = 23 individuals with CT or TT genotypes). Relative levels of each mRNA expression were obtained after normalization to GAPDH. Then, percentile change is obtained respective to an average of β-*NRXN3* mRNA in control individuals with CC genotype. *P < 0.05, **P < 0.001, ***P < 0.0001, calculated using two-tailed Mann-Whitney tests

The most prominent result of these assays, as with studies of total *NRXN3*, α-*NRXN3*, and β-*NRXN3* mRNA levels, was the reduced expression that was found for most of the isoforms in the AD postmortem middle frontal gyrus (MFG), middle temporal gyrus (MTG), and hippocampus (HIP) (Fig. 3c). In comparison with one-half reduction of total *NRXN3* expression in the AD cortex, the exon 22a-24b variant that encodes the major transmembrane isoform was expressed at levels that were decreased, by 85% in AD subjects with either CC or CC/TT genotypes (*P* < 0.0001 by two-tailed Mann-Whitney tests for both comparisons) (Fig. 4b). Levels of the exon 22a-24c variant, the second major transmembrane isoform were also decreased by 56% and 66% in AD patients with CC and with CC/TT genotypes when compared with control CC individuals (Fig. 4c; *P* < 0.0001 by two-tailed Mann-Whitney test for both comparisons). Overall differences in mRNA expression levels for these two transmembrane isoforms between CC and CT/TT genotypes were not detected among the AD patients (*P* = 0.611 and 0.476 by two-tailed Mann-Whitney tests, respectively).

The major soluble *NRXN3* isoform, encoded by the exon 22a-23a variant mRNA was also decreased by 63% and 71% in AD patients with CC and CC/TT genotypes when compared with those in control individuals with CC genotypes (Fig. 4d; *P* < 0.0001 by two-tailed Mann-Whitney test for both comparisons). Differences in mRNA expression levels between CC and CT/TT genotypes were not detected among the AD brains (*P* = 0.455 by two-tailed Mann-Whitney test).

**Fig. 5:**
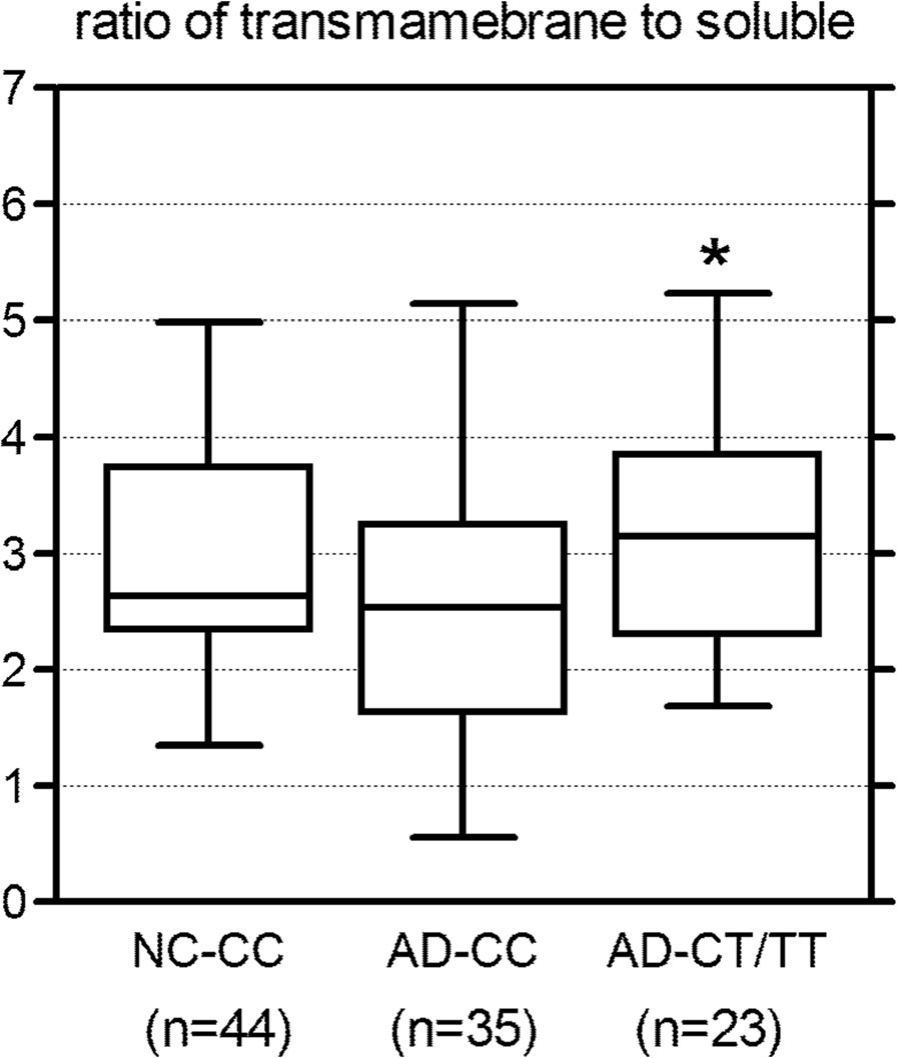
Ratios of expression levels in total transmembrane isoforms to soluble isoform. In AD groups, ratios of transmembrane to soluble isoform show a significant difference between individuals with CC genotype and those with CT or TT genotypes. The ratios were increased by 25% in AD patients with CT or TT genotypes compared with AD with CC genotype (*P = 0.053 by two-tailed Mann-Whitney test and *P* = 0.044 by unpaired *t* test

Closer examination revealed evidence for interactions between the clinical phenotype, the AD susceptible rs8019381 T allele, and expression of the exon 22a-24a mRNA that encodes a major transmembrane isoform (Fig. 4a). Expression of exon 22a-24a mRNA was decreased by 46% in AD patients with CT/TT genotypes when compared with those in control individuals with CC genotypes (*P* = 0.0002, two-tailed Mann-Whitney tests). Within the AD group, exon 22a-24a mRNA expression levels were also decreased by 46% in AD patients with CT/TT genotypes when compared with those with CC genotypes (*P* = 0.043 by two-tailed Mann-Whitney tests). By contrast, exon 22a-24a mRNA expression levels did not differ significantly between control and AD samples with CC genotypes (*P* = 0.180 by two-tailed Mann-Whitney test). These differences correlated with differences in the ratios between transmembrane and soluble isoform expression in CC vs CT/TT AD individuals, and the ratio differences reached the margin of statistical significance (Fig. 5). In AD patients, the ratios of transmembrane vs soluble isoforms were 25% greater in CT/TT than in CC subjects (*P* = 0.053 by two-tailed Mann-Whitney test and *P* = 0.044 by unpaired *t* test) despite that the overall ratio of the transmembrane and soluble isoforms decreased in AD brains. Interestingly, these ratios did not differ between AD patients with CT/TT genotypes vs those in control CC individuals (*P* = 0.331 by two-tailed Mann-Whitney tests).

### Inverse correlation of *NRXN3* with inflammasome component *NLRP3* in AD brains

**Fig. 6:**
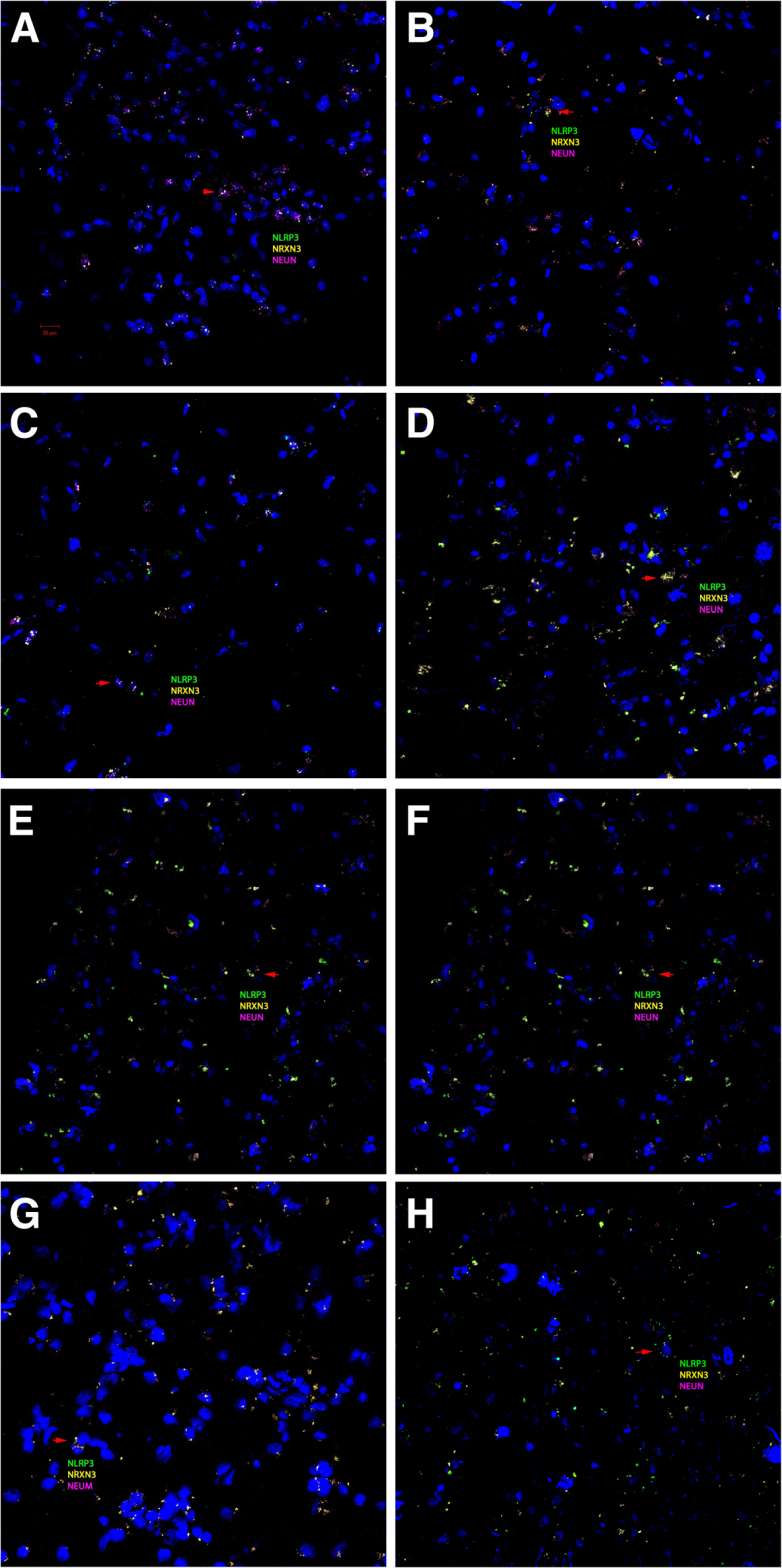
**a**–**h** RNAscope in situ hybridization of control and AD brain samples with different Braak numbers Green represents *NLRP3*, yellow *NRXN3*, and magenta *NEUN*. The red arrow indicates colocalization of three probes in the same cell. H-score correlations of *NRXN3* and *NLRP3* intensities with Braak numbers. (**a**) MTG-Braak 0; (**b**) MTG-Braak 1; (**c**) MTG-Braak 2; (**d**) MTG-Braak 4; (**e**) MTG-Braak 5; (**f**) MTG-Braak 6; (**g**) HIP-Braak 0; (**h**) HIP-Braak 6

**Fig. 7:**
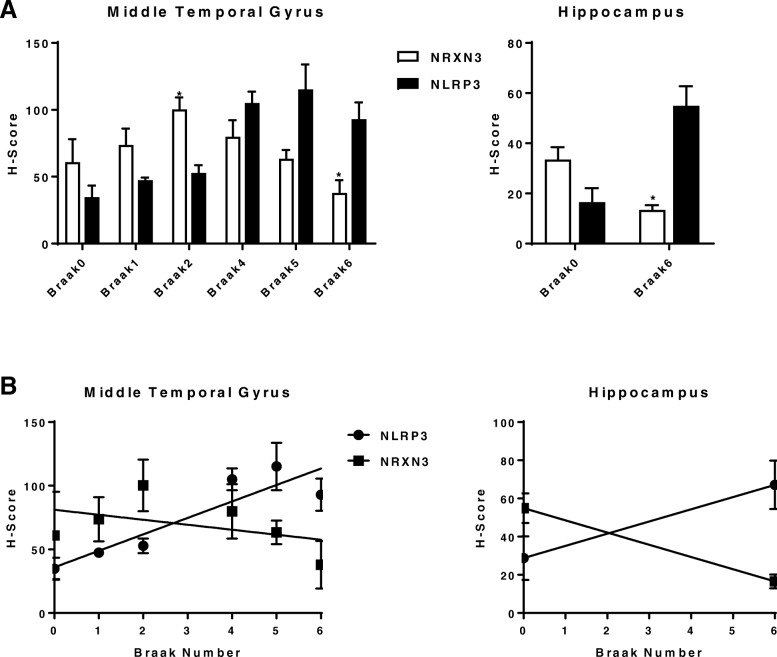
**a** Neuron expression of *NRXN3* and *NLRP3* in MTG and HIP of different Braak stages. **b** Linear regression of H-scores (*Y*-axis) of *NRXN3* and *NLRP3* with Braak numbers (*X*-axis) for MTG and HIP

We carried out an ultra-sensitive RNAscope ISH assay to study the altered *NRXN3* expression at cellular levels in control and AD postmortem brain samples that were co-hybridized and co-stained with inflammasome component *NLRP3* [41] and neuron marker *NEUN*/*RBFOX3*. All *NRXN3* and the majority of *NLRP3* signals were co-localized with *NEUN*/*RBFOX3-*positive neurons (Fig. 6). We found that the reduced *NRXN3* mRNA was inversely correlated with the increased *NLRP3* mRNA in *NEUN*/*RBFOX3*-positive neurons of the AD middle temporal gyrus (Fig. 6a–f) and hippocampus (Fig. 6g, h) samples. Two-tailed and unpaired Student’s *t* test using H-scores that represent *NRXN3* neuron expression was significantly higher than that of *NLRP3* at Braak 2 stage of MTG and significantly lower at Braak 6 stage in MTG and HIP (Fig. 7a). Two-way ANOVA analysis found significant differences of H-scores in HIP (*F*_1,14_ = 6.07; *P* = 0.0273) but not in MTG (*F*_1,28_ = 0.45; *P* = 0.4515) with different Braak stages; however, the interaction of *NRXN3* and *NLRP3* neuron expression in MTG and HIP at different Braak stages were very significant (*P* < 0.0001). Linear regression analysis using H-score for each Braak number (0, 1, 2, 4, 5, 6) found that the differences of slopes of *NRXN3* and *NLRP3* were significant (*F*_1,8_ = 11.49; *P* = 0.0095) in MTG samples. *NLRP3* regression slope was significantly non-zero (*F*_1,4_ = 18.32; *P* = 0.0128), and *NRXN3* regression slope was not significantly non-zero (*F*_1,4_ = 0.98; *P* = 0.3773). The correlation was also simulated in control (Braak = 0) and AD (Braak = 6) in HIP samples. The linear regression lines for *NRXN3* and *NLRP3* intersected at 2.6 and 2.1 Braak grades in MTG and HIP samples, respectively (Fig. 7b). We did not observe any significant correlation of *NEUN*/*RBFOX3* with Braak stages (data not shown).

## Discussion

We found that *NRXN3* gene haplotype interacts with the *APOE* ε4 haplotype, and the expression and ratio of its transmembrane and soluble isoforms were reduced in AD postmortem MFG. *NRXN3* mRNA level was inversely correlated with that of inflammasome component *NLRP3* in MTG AD neurons. The linear regression of *NRXN3* and *NLRP3* signals that intersected at Braak 2.1 for HIP and Braak 2.6 for MTG might indicate differential progression of Aβ fibrils in different brain regions. Previous studies by array tomography and electron microscopy find that AβO forms halo at synapses that attracts Aβ fibrils around damaged neurites [42–44]. The most prominent AD-associated susceptible genes and their altered expression/splicing/translation/PTM (posttranslational modifications), such as *APP* and *PSEN1* [23, 45], *APOE* and *APOER2* [46], *PTK2B* [47], *PPP3CA* and *PPP3R1* [48, 49], and *PIN1* [50], are involved in synaptic homeostasis. Dysregulation of presynaptic *NRXN3* might be an early event that triggers synaptic calcium dyshomeostasis and let AβO invasion at synapses. Subsequent dystrophic neurites and dysfunctional synapses stimulate *NLRP3*/caspase-1 and calcineurin/caspase-3 pathways that activate interleukin-1β and interleukin-18 [51, 52] and cause mitochondria impairment and apoptosis [53], respectively. Aβ fibrils are at their peak when AD symptom just appears [54], and *NRXN3* and *NLRP3* expression trajectories might serve as early diagnosis and therapeutic targets at early Braak 2–3 stages.

*NRXN3* SNP rs8019381 was found to contribute to AD susceptibility. There is no information available for rs8019381 in previously reported genome-wide association studies for AD [3, 55, 56] because of the relatively small haplotype block (14 kb) in the study. While no genomic markers that display strong linkage disequilibrium with rs8019381 are identified in the Translational Genomic Research Institute (TGen) datasets [56], we identified rs2067730 that lies about 6 kb 5′ to rs8019381 in genome-wide association studies of clinically diagnosed AD vs control subjects of European ancestries who were recruited from Canadian memory clinics [55]. Interestingly, like *NRXN3* rs801938, rs2067730 displays association with AD in this sample and appears to interact with the *APOE* genotype (*P* = 0.027).

The magnitude of rs8019381 association suggests an odds ratio of 2.48, with a broad 95% confidence interval that encompasses 1.6–3.9. While this effect is much less than the large, oligogenic influence of *APOE* haplotypes on AD vulnerability, it is larger than many of the effects of other proposed polygenic variants listed in systematic meta-analysis presented on the AlzGene database [57] or in two genome-wide association datasets that compare AD vs control samples [3, 55, 56]. The effects of the *NRXN3* haplotype may be even larger in individuals with specific *APOE* haplotypes. Both the current dataset and data reported by Li et al [55] provide evidence for significant interactions among *APOE* haplotypes and 3′ *NRXN3* haplotypes in AD.

The reduced expression of total *NRXN3*, α-*NRXN3*, and β-*NRXN3* in samples of the cerebral cortex and hippocampus from pathologically confirmed AD and control brains formed the initial basis for implicating *NRXN3* in AD. These findings were accompanied by a significant reduction of ratios between transmembrane and soluble isoforms in AD individuals with rs8019381 CT or TT genotypes. These observations support the hypothesis that reduction of *NRXN3* transmembrane isoform alters synapse homeostasis, reduces neurotransmitter release, and promotes Aβ oligomerization and *APOE* dysfunction in synaptic degeneration [7, 58]. Alternatively, the altered ratio might differentially interact with alternatively spliced isoforms of *APP,* causing increased Aβ production [59].

The associated *NRXN3* SNP rs8019381 is located at the junction of exon 23’s splicing donor site (23 base pairs downstream from the 3′ of exon 23), within a region that might alter splicing efficiency. The *NRXN3* haplotype studied here is likely to be different from other ethnic samples. The rs8019381 “T” allele frequency (0.07) of the control samples reported here (based on genotypes from 672 chromosomes) is similar to values obtained in unselected Europeans and European-Americans available (0.094 based on genotypes from 224 chromosomes) from dbSNP. African Yoruban rs8019381 frequency (0.198) is (based on genotypes of 180 chromosomes) higher than European population, and East Asian rs8019381 frequency is much lower than European and African populations, with none of the “T” allele detected in Chinese (based on genotypes of 90 chromosomes) and 0.006 detected in Japanese (based on genotypes of 172 chromosomes) HapMap samples. Additional studies will be necessary to identify more informative *NRXN3* rs8019381 for use in individuals with non-European heritage. Much of the evidence presented here provides an increased focus on the role of synaptic pathology in AD. Synapse losses can be documented with the first clear-cut evidence for dementia that are accompanied by synaptic toxicities conferred by *APP* [60] and *APOE* mutations [61]. The evidence in the current report suggests that Aβ and *APOE* synaptic pathologies are likely to interact with allele-specific alterations in gene expression of *NRXN3* transmembrane and soluble isoforms.

An astronomical number of synapses derived from about 86 billion human brain neurons [62] are dynamic throughout the human life span, and their damage precedes neuron death due to Aβ oligomer (AβΟ) toxicity in AD [18, 63]. Substantial microscopic Aβ plaques are observed in old adult brains with intact cognition function [6]; however, nano-synaptic-space distribution of AβO is less known and neurexin complexes are known partners of Aβ [8]. Complex neurexin alternative splicing codes define synaptic specificity, strength, plasticity [15, 31], and vulnerability toward AβO [64]. The trans-synaptic anterograde and retrograde signaling of the neurexin-neuroligin-endocannabinoid system [13, 31, 65, 66] provides an attractive pathway for AD therapeutic development. Modulation of presynaptic and postsynaptic endocannabinoid tone through CB1R [67] and CB2R [68, 69], respectively, by their specific ligands might reduce neuron inflammasome and shift neurexin-neuroligin alternative splicing repertoire toward heathy synapses and reverse cognitive decline during aging [70, 71]. Neurexin peptides are significantly elevated in cerebrospinal fluid (CSF) of individuals with mild cognitive impairment (MCI), especially in patients with MCI progressing to AD dementia [72, 73]. Conceptually, targeted tryptic peptide panels of specific neurexin isoforms will improve CSF early diagnosis for pre-symptomatic AD. Screening of effective neurexin and cannabinoid receptor ligands [66, 74] and behavioral modulation of mental activities and nutrition intakes [75] might help to improve synaptic health and prevent cognitive decline 10 years or more before AD symptom appearance.

## Conclusion

*NRXN3* rs8019381 SNP located at SS#5 splicing site was found to contribute to AD susceptibility and interact with the *APOE* ε4 haplotype. The altered expressions of *NRXN3* transmembrane and soluble isoforms were further reduced in susceptible rs8019381 heterozygous and homozygous alleles (CT or TT) in the AD postmortem cortex. The reduced *NRXN3* expression was inversely correlated with the increase of inflammasome component *NLRP3* expression in *NEUN*/*RBFOX3*-positive neurons in the AD brain.

## Abbreviations

AD: Alzheimer’s disease
Aβ: Amyloid beta
AβO: Amyloid beta oligomer
CSF: Cerebral spinal fluid
HIP: Hippocampus
HWE: Hardy-Weinberg equilibrium
LNS: Lamin-neurexin-sex hormone-binding globulin domains
MCI: Mild cognitive impairment
MFG: Middle frontal gyrus
MTG: Middle temporal gyrus
NFT: Neurofibrillary tangles
PCR-RFLP: PCR and restriction fragment length polymorphism
PDZ: *PSD95*-*Dlg1*-*Zo1* domain
PMI: Postmortem interval
PTM: Posttranslational modification
RT-PCR: Reverse transcription polymerase chain reaction
SNP: Single nucleotide polymorphism
SS#5: Splicing site 5
