# Correction to: Neurexin 3 transmembrane and soluble isoform expression and splicing haplotype are associated with neuron inflammasome and Alzheimer’s disease

**DOI:** 10.1186/s13195-019-0493-0

**Published:** 2019-05-07

**Authors:** Akitoyo Hishimoto, Olga Pletnikova, Doyle Lu Lang, Juan C. Troncoso, Josephine M. Egan, Qing-Rong Liu

**Affiliations:** 1 Department of Psychiatry, Kobe University Graduate School of Medicine, 7-5-1 Kusunoki-Cho, Chuo-Ku, Kobe, 650-0017 Japan; 2 Departments of Pathology, Neuropathology Division, Johns Hopkins University School of Medicine, 600 North Wolfe Street, Baltimore, MD 21205 USA; 3 Lab of Clinical Investigation, NIA-NIH, 251 Bayview Blvd, Baltimore, MD 21224 USA

**Correction to: Alzheimers Res Ther (2019) 11:28**

**https://doi.org/10.1186/s13195-019-0475-2**

Following publication of the original article [1], the authors reported that Fig. 6 contains a mistake. The Fig. 6f is a duplicate of Fig. 6e of Braak 5.

The correct Fig. 6f is shown below.

**Fig. 6:**
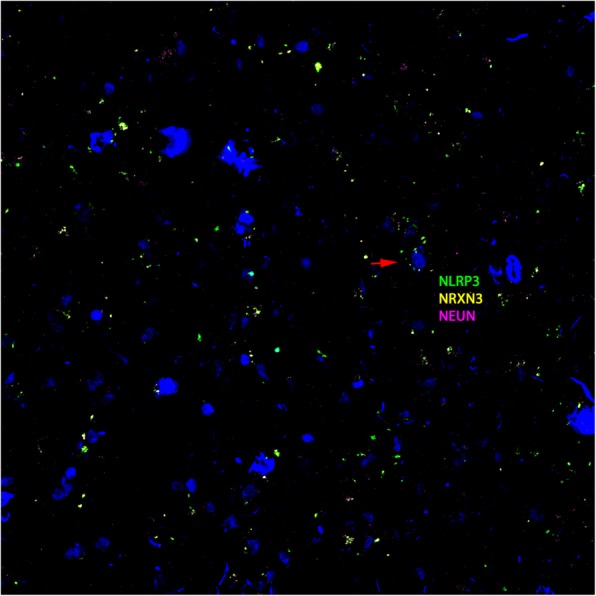
**a**–**h** RNAscope in situ hybridization of control and AD brain samples with different BRAAK numbers (**a**). Green represents NLRP3, yellow NRXN3, and magenta NEUN. The red arrow indicates colocalization of three probes in the same cell. H-score correlations of NRXN3 and NLRP3 intensities with BRAAK numbers (**b**)
